# Radiosensitizers in cervical cancer. Cisplatin and beyond

**DOI:** 10.1186/1748-717X-1-15

**Published:** 2006-05-08

**Authors:** Myrna Candelaria, Alicia Garcia-Arias, Lucely Cetina, Alfonso Dueñas-Gonzalez

**Affiliations:** 1 Division of Clinical Research, Instituto Nacional de Cancerología, Mexico; 2 Unidad de Investigación Biomédica en Cancer, Instituto Nacional de Cancerología/Instituto de Investigaciones Biomedicas, UNAM, Mexico

## Abstract

Cervical cancer continues to be a significant health burden worldwide. Globally, the majority of cancers are locally advanced at diagnosis; hence, radiation remains the most frequently used therapeutical modality. Currently, the value of adding cisplatin or cisplatin-based chemotherapy to radiation for treatment of locally advanced cervical cancer is strongly supported by randomized studies and meta-analyses. Nevertheless, despite these significant achievements, therapeutic results are far from optimal; thus, novel therapies need to be assayed. A strategy currently being investigated is the use of newer radiosensitizers alone or in combination with platinum compounds. In the present work, we present preclinical information on known and newer cytotoxic agents as radiosensitizers on cervical cancer models, as well as the clinical information emanating from early phase trials that incorporate them to the cervical cancer management. In addition, we present the perspectives on the combined approach of radiation therapy and molecular target-based drugs with proven radiosensitizing capacity.

## Epidemiology and cervical cancer treatment

Cervical cancer remains one of the greatest killers of women worldwide. According to Globocan 2000, it is estimated that in 2000 the numbers of patients diagnosed with and those who died from this disease were 470,606 and 233,372, respectively [1]. It is remarkable that these rates occur, despite the fact that cervical cancer is a model for early detection due to its long and relatively well-known natural history that offers an excellent opportunity for its detection before lesions become invasive [2].

Cervical cancer is currently staged clinically according International Federation of Gynecology and Obstetrics (FIGO) guidelines. In terms of treatment, invasive disease can be divided into three main groups: 1) early stage, which ranges from microinvasive disease IA1, IA2 to macroscopic disease confined to cervix and measuring <4 cm, IB1; 2) locally advanced FIGO stages IB2-IVA, and 3) IVB and recurrent disease [3].

### Treatment of early stage cervical cancer

The recommended treatment for IA1 patients is either a local procedure such as conization or total hysterectomy, depending on the patient's desire to remain fertile, whereas for IA2 patients the recommendation is for a radical hysterectomy which removes parametrial tissue, upper vagina and pelvic lymph nodes. On average, 8% of cases show positive pelvic lymph nodes. Because many women at this disease stage wish and deserve to preserve fertility, radical trachelectomy is becoming an option for these patients as well as for IB1 patients [4]. In surgically treated early-stage cases, the presence in the surgical specimen of a combination of intermediate-risk factors (vascular and lymphatic permeation, tumor size >2 cm, and deep cervical stroma invasion) or high-risk factors (positive pelvic lymph nodes, parametrial infiltration, and positive surgical margins) dictates the use of adjuvant radiation [5] or chemoradiation, respectively [6].

### Treatment of locally advanced stages

Treatment results for these patients are far from optimal. In this regard, treatment of locally advanced cervical cancer experienced no major changes for the nearly 80 years during which exclusive radiation was considered the standard of care; thus, 5-year survival for stages IB2, IIB, IIIB, and IVA are 72.2, 63.7, 41.7, and 16.4%, respectively, according to the 1998 Annual Report on the Results of Treatment in Gynecological Cancer [7]. The lengthy permanence of this unimodal treatment was due, on the one hand, to the classical concept that cervical cancer is a disease that progresses in an orderly fashion (local, then regional, and at the very last, systemic); therefore, it could be effectively treated with a local modality such as radiation instead of a systemic modality such as chemotherapy. On the other hand, the role of surgery for locally advanced cases failed to treat the disease successfully by radical surgical procedures [8].

Over the last 20 years, a number of trials testing concurrent chemoradiation were performed in an attempt to improve treatment results. Despite this, in 1996 a National Institutes of Health Consensus Statement on cervical cancer stated that there was no evidence that hydroxyurea or any other concomitant chemotherapeutic agent should be added to pelvic irradiation and incorporated into standard practice [9]. It was not until 1999 that five randomized studies including nearly 2,000 patients were published, demonstrating that survival rate with concomitant chemotherapy (RT/CT) based on cisplatin was superior than that obtained with radiation alone [6,10-13]. Afterwards, a meta-analysis based on 19 trials (17 published and two unpublished) including 4,580 patients corroborated these findings, confirming that chemoradiation offers an absolute survival benefit of 12% at 5 years [14]. Thus, cisplatin-based chemoradiation was largely accepted as the standard of care for patients with cervical cancer whose treatment required radiation, except for patients with co-morbidities who are radiated for stage IB1 or less. An update of the aforementioned meta-analysis that includes 24 trials (21 published, three unpublished) and 4,921 patients strongly suggests that chemoradiation improves overall survival and progression-free survival, whether or not platinum was used, with absolute benefits of 10 and 13%, respectively. There was, however, statistical heterogeneity for these outcomes. There was some evidence that the effect was greater in trials including a high proportion of stage I and II patients. Chemoradiation also showed significant benefit for local recurrence and the suggestion of a benefit for distant recurrence. Acute hematological and gastrointestinal toxicity was significantly higher in the concomitant chemoradiation group. Treatment-related deaths were rare, but late effects of treatment were not well-reported; thus, the impact of chemoradiation on these effects could not be determined adequately [15].

## Standard cisplatin-based chemoradiation. The role of hydroxyurea and 5-fluorouracil

### Hydroxyurea

Three of the five National Cancer Institute (NCI)-sponsored studies that served for recommending chemoradiation as standard of care for all patients with locally advanced cervical cancer whose treatment require radiation used oral hydroxyurea in the control arm. This agent is a cytotoxic drug first used in clinical trials in the late 1960s that has properties that suggest it could be a good radiosensitizer; for instance, it inhibits ribonucleotide reductase and induces a block at the G1-S phase of the cell cycle when cells are particularly sensitive to radiation, prevents repair of sub-lethal radiation damage, and increases the killing of cells under hypoxic conditions. Its major side effect is myelosuppression [16]. Early trials published by the Roswell Park suggested the usefulness of hydroxyurea as radiosensitizer for cervical cancer [16]. Therefore, the Gynecologic Oncology Group (GOG) randomized patients with stage IIIB or IVA squamous cell carcinoma of the cervix to receive either standard radiotherapy or radiation and concurrent hydroxyurea [42]. The hydroxyurea arm resulted in a higher complete response rate (68 vs. 49%), longer median progression-free and median survival (13.6 vs. 7.6 m) and (19.5 vs. 10.7 m), respectively. Nonetheless, because one half of randomized patients were unevaluable and were thus excluded from analysis, among other study defects, the findings of this study were questionable [17]. In the subsequent GOG 56 protocol, 296 patients with surgically evaluated node-negative stage IIB-IVA stages were randomized to receive either concurrent hydroxyurea and radiation or the hypoxic cell sensitizer misonidazole and radiotherapy. On subgroup analysis, patients with stage IIIB or IVA disease who received hydroxyurea were found to have a statistically significant improvement in progression-free interval (42.9 vs. 40.4 m) but not in survival, compared with those who received misonidazole [18].

Based on the results of this study, the GOG adopted the hydroxyurea and radiation combination as standard therapy; nevertheless, this treatment was not widely adopted outside of the GOG, and particularly in Europe. Further, the concern that the observed difference in GOG 56 was due to reduced survival associated with use of misonidazole rather than additional benefit from hydroxyurea was supported by the results of RTOG 8O-05, which randomized 120 patients with stages IIIB and IVA squamous cell carcinoma of the cervix to receive either standard radiation or standard radiation and the hypoxic cell sensitizer used in GOG 56, misonidazole. Patients who received misonidazole had similar or slightly worse survival than conventional radiotherapy-treated patients [19]. These results were updated, observing 10-year overall survival for patients in the misonidazole arm of 45%, compared with 49% for the placebo arm (*p *= 0.89). It is noteworthy that 10 patients (14%) developed severe late complications and the 10-year serious-late-complication rate was 14 and 12% for experimental and control arms, respectively (*p *= 0.51) [20]. A recent systematic review critically evaluating all published randomized control trials concluded with lack of convincing evidence to suggest a therapeutic effect of hydroxyurea, supporting the exclusion of this drug from current or further chemoradiotherapy schedules [16].

### 5-Fluorouracil

As opposed to gastrointestinal cancers [21], single agent 5-fluorouracil (5-FU) has not been widely evaluated as a radiosensitizer in cervical cancer. The sole previous randomized trial of FU concurrent with RT for cervix cancer included 234 women with bulky IB-IVA cervix cancer randomized to either standard radiotherapy with or without a 4-day infusion of 1 gr/m^2 ^of 5-FU on days 1–5 and 22–25 or partially hyperfractionated radiation with or without the same chemotherapy regimen. Standard radiation consisted of a combination of external beam and intracavitary radiation to deliver 90 Gy to Point A, the partially hyper-fractionated regimen delivered two radiation fractions, 6 hours apart, on the first 4 and last 4 treatment days, coinciding with 5-FU infusion. Results show that addition of 5-FU did not improve pelvic control or overall survival; nonetheless, the study was underpowered due to early closing. In subset analysis, there was a statistically significant improvement in disease-free survival for patients with stages IB, IIA, or medial parametrial IIB disease receiving concurrent standard radiotherapy and 5-FU [22].

Two of the five randomized trials demonstrated the superiority of combined chemotherapy with radiation in advanced cervix cancer treatment when the cisplatin/fluorouracil (FU) combination was used [10,13]. In the GOG 120, weekly cisplatin was directly compared with FU/cisplatin/hydroxyurea in combination with radiation. While there was no difference in progression-free survival between cisplatin alone and the FU-cisplatin regimen, a significant difference in adverse events for the three-drug regimen was demonstrated [13]. Thus, the regimen of weekly cisplatin concurrent with radiation was chosen as the standard treatment approach in the GOG for locally advanced cervix cancer and was the regimen with which new combination treatments should be compared. FU when used by protracted venous infusion (PVI) compares favorably with bolus FU in combination with radiation in high-risk rectal cancer, demonstrating an improvement in relapse-free and overall survival [23]. Therefore, this modality seems appealing for cervix cancer treatment because increased drug-radiation interactions with tumor cells should occur with potentially increased radiation sensitivity and pelvic control. The disadvantage of this approach comprises the need for central access and specialized equipment to deliver continuous FU, and the potential for increased acute and chronic bowel toxicity. These data led the GOG to perform a phase III trial of standard weekly cisplatin in combination with radiation, comparing with the experimental arm of protracted venous infusion of FU (GOG 165). The study was performed in patients with stage IIB, IIIB, and IVA cervical cancer with clinically negative aortic nodes. The pelvic dose of radiation was 45 Gy with a parametrial boost to involved sides of 5.4–9 Gy, and high- or low-dose rate intracavitary brachytherapy. The standard regimen was weekly cisplatin 40 mg/m^2^, and experimental therapy was PVI FU 225 mg/m^2^/d for 5 d/wk for six cycles during RT. The study was closed prematurely when a planned interim futility analysis indicated that PVI FU/RT had a higher treatment failure rate (35% higher) and would, most likely, not result in improvement in progression-free survival compared with weekly cisplatin/RT [24]. Taken together, existing information suggests that 5FU alone has not major role when used as radiosensitizer in treatment of cervical cancer treatment.

## Platinum compounds as radiosensitizers in cervical cancer. Alternative schedules and agents

### Cisplatin

Although it is widely accepted that cisplatin-based chemoradiation is the standard treatment for locally advanced cervical carcinoma, optimal scheduling and dosing have yet to be established. Evidence from the GOG125 study indicates that weekly cisplatin at 40 mg/m^2 ^for six weeks is equally effective yet less toxic than cisplatin and 5-fluorouracil in a classic 21-day schedule [13]; nonetheless, the choice of 40 mg/m^2 ^as the dose for weekly cisplatin for phase III chemoradiation trials was not based on previous phase I data, and the maximum tolerated dose of weekly cisplatin in combination with pelvic radiation has not been clearly defined. However, indirect data from subsequent studies of chemoradiation in non-protocol settings suggest that this dose of cisplatin is perhaps the maximum tolerated. For instance, Abu-Rustum et al. reported on 65 women from minorities (African-American, Caucasian, and Hispanic) receiving weekly cisplatin during radiation; overall, 19 of 65 (29.2%) patients had incomplete chemotherapy, nine due to hematological or renal toxicity. Thus, only seven patients (10.8%) received six cycles of cisplatin, although the majority (60%) received five applications [25]. In another report, 112 patients with cervical cancer received five planned courses of cisplatin at 40 mg/m^2 ^during external radiation; 62 patients (55%) did not undergo the five planned cycles of cisplatin due to treatment toxicity (31%) or non-compliance due to delayed first-cycle administration or omission of a cycle for reasons other than toxicity (21%) [26].

In our experience, under this dose of weekly cisplatin during external beam radiation only 67% of patients receive the six planned courses of weekly cisplatin [27]. Nonetheless, as stated previously, optimal scheduling and dosing of cisplatin to be used in cervical cancer radiosenzitation have not yet been established. There are indications that timing between cisplatin administration and irradiation significantly influences results. The largest therapeutic effect was observed when the drug was administered daily prior to each irradiation fraction, as demonstrated in lung cancer. In 1992, the Radiotherapy and Lung Cancer Cooperative Groups of the European Organization for Research and Treatment of Cancer (EORTC) reported the results of a randomized phase III study of concomitant cisplatin and radiotherapy vs. radiotherapy alone in patients with non-metastatic, inoperable, non-small cell lung cancer that accrued 331 patients (70% of whom had squamous cell cancer). Patients were randomly assigned to radiotherapy alone (group 1), the same radiotherapy combined with cisplatin at a dose of 30 mg/m^2 ^by intravenous (i.v.) infusion on the first day of each treatment week (group 2), or cisplatin at a dose of 6 mg/m^2 ^administered daily 1–2 h prior to radiotherapy (group 3). The largest and significant benefit was seen in the radiotherapy/daily cisplatin group. Both survival and control of local disease were significantly improved, compared with the radiotherapy-only group (*p *= 0.009 and 0.003, respectively) [28].

In cervical cancer, a group from Japan reported on the results of a phase I study of daily cisplatin and concurrent radiotherapy in which 14 patients with locally advanced cervical carcinoma and 13 who required postoperative RT were registered. A low dose of cisplatin was given daily concurrently with RT. Cisplatin dosing was initiated at 6 mg/m^2^/day, which was incremented by 0.5 mg/m^2^/day. Radiation was delivered at 2 Gy/day to a total dose of 50 Gy. Maximum Tolerated Dose (MTD) was defined as the dose level immediately below that causing dose-limiting toxicity (DLT) in over one third of treated patients. In 88% of patients (22 of 25), cisplatin was administered continuously as planned without interruption (the MTD, 8 mg/m^2^. Interestingly, this daily dose of cisplatin is roughly equivalent to the weekly dose of 40 mg/m^2 ^[29]. Thus, although daily cisplatin administration has the potential to improve results, the approach possesses practical limitations that render its wide adoption unlikely.

### Carboplatin

Carboplatin and cisplatin have an identical mechanism of cytotoxicity. The quantitative level of platinum DNA adduct formation correlates with the degree of cell killing [30]. Carboplatin has a greater degree of chemical stability; as a consequence, a higher dose of carboplatin is necessary to obtain comparable antitumor effect in experimental systems. Thus, the therapeutic carboplatin dose relative to the therapeutic cisplatin dose has been described as a ratio of 4:1 (400–500 mg/m^2 ^vs. 100 mg/m^2^, based on clinical studies in ovarian cancer [31]. Like cisplatin, carboplatin is an effective radiosensitizer in a variety of *in vitro *and *in vivo *systems, targeting the hypoxic cell population as well as potentiating cell kill after irradiation [32,33].

Despite the fact that weekly cisplatin during radiation is well-tolerated, its nephrotoxicity is of particular concern in a patient population that frequently harbor renal dysfunction as a consequence of ureteral obstruction by the disease spreading to the pelvic wall or to the bladder. Although <10% of patients with bulky tumors with uni- or bilateral ureteral obstruction present abnormal creatinine serum levels [34], subclinical changes in renal function are known to occur in renal obstructive disease [35]. Moreover, these patients are exposed to iodinated contrast media during (i.v.) pyelography and/or computed tomography (CT) scan, which also induce subclinical renal dysfunction [36]. These data situate carboplatin, devoid of renal toxicity, as an alternative for radiosensitization in cervical carcinoma. There are some reports on use of carboplatin concurrently to radiation.

Micheletti et al. reported a phase I-II study of radiation plus continuous infusion of carboplatin in 12 patients with stages IIB and IIIB cervical cancer, using 12 mg/m^2^/day for a total dose of 504 mg/m^2 ^in 42 days, equivalent to 250 mg/m^2 ^every 21 days for two courses. This schedule proved well-tolerated and effective, leukopenia grade 2 the most frequent toxicity and complete response was observed in 75% of patients. Interestingly, patient pharmacokinetic studies showed that the platinum steady-stated in both plasma and tumor cells was not achieved and was below the concentration required *in vitro *to produce radiopotentiation; these results suggested that the optimum dose of carboplatin must be higher [37]. In another report on carboplatin as radiosensitizer, 22 patients staged from IIA-IIIB were treated with 30 mg/m^2 ^twice a week with escalation at 40 mg/m^2 ^and 50 mg/m^2^; however, after several patients were treated the dose was re-calculated according to area under the curve (AUC). Accordingly, the authors suggest that an AUC of 6 could be adequate on the basis that only two of nine patients presented leukopenia grade 3 [38]. In a phase I study of weekly carboplatin during radiation, 24 FIGO stage IIIB patients were treated with standard pelvic radiation concurrently with six weekly applications of carboplatin at the following dose levels: I (100 mg/m^2^); II (116 mg/m^2^); III (133 mg/m^2^), and IV (150 mg/m^2^). Six patients per level were treated, and all but two completed external beam and intracavitary treatment. The treatment was well-tolerated, median number of weekly applications of carboplatin was six, and dose-limiting toxicity (leukopenia and/or neutropenia) was present in 50% of patients treated at the higher-dose level (150 mg/m^2^), whereas this occurred in 33% of patients at 133 mg/m^2^; hence, this dose was that recommended for use in further trials. Remarkably, the clinical response rate was similar to that reported for standard cisplatin [39].

### Nedaplatin

Nedaplatin (254-S) is an investigational platinum analog developed in Japan that possesses a novel structure involving a glycolate ring bound to the platinum atom as a bidentate ligand. The agent has pronounced preclinical antitumor activity and virtual lack of nephrotoxicity with a clinical spectrum similar to that of cisplatin [40]. *In vitro *cytotoxicity of nedaplatin against fresh cervical cancers from untreated patients indicates that it is an active agent with similar activity to those of cisplatin and carboplatin [41]. A small study of nedaplatin as radiosensitizer has been carried out in 10 cervical cancer patients used at 70 mg/m^2 ^on days 1 and 29. The treatment was well-tolerated, the main toxicity being hematological [42].

### Oxaliplatin

Although oxaliplatin has demonstrated radiosensitizing properties in experimental mice models [43] and appeared to be a better radiosensitizer than 5FU in colon carcinoma cell lines [44], its use in combination with radiation has to date only been explored in locally advanced rectal cancer along with 5-fluorouracil, where it has proven to be active and well-tolerated [45]. In cervical cancer, clinical antitumor activity either alone or in combination with other agents has been demonstrated [46,47], but no trials have reported its use as radiosensitizer.

## Older radiosensitizer agents

### Mitomycin C

Mitomycin C (MMC) is a naturally occurring anti-tumor quinone derived from Streptomyces caespitosus, an actinomyces strain. It has been used as a cytotoxic since the 1960s and possesses activity against lung, stomach, head and neck, prostate, breast, and bladder tumors. It is regarded as the prototypical bioreductive drug, as it is inactive until reduced by one-electron reductases or DTD [48]. Reduction allows DNA cross-links and strand breaks to form, thus inhibiting DNA replication. MMC sensitivity has been shown associated with high DTD levels by some workers, but others have demonstrated discrepancies between *in vitro *and *in vivo *cytotoxicity. Relating MMC cytotoxicity to DTD levels is complicated by the fact that MMC is a relatively poor substrate for DTD, and is also bioactivated by one-electron reductases; activation is pH-dependent and may be influenced by hypoxia [49]. Mitomycin C is a well-known radiosensitizer whose main indication continues to be treatment of locally advanced anal cancer in combination with 5-fluorouracil and radiation [50].

In cervical cancer, the majority of studies with chemoradiation utilize mitomycin in combination with 5-fluorouracil. Early studies of this combination employing several doses and schemes of 5-fluorouracil and mitomycin C were small and included patients with advanced and recurrent disease. Results were reported as encouraging, with good control local rates and tolerable side effects [51-53]. The efficacy of this combination was further supported by the results of a prospective, phase III multicenter randomized trial to assess the effectiveness of concurrent i.v. mitomycin C, oral 5-fluorouracil, and radiotherapy in locally advanced carcinoma of the cervix. This study included 926 FIGO Stage IIB-IVA patients who were randomized into four arms: arm 1: conventional RT; arm 2: conventional RT and adjuvant chemotherapy; arm 3: conventional RT plus concurrent chemotherapy, and arm 4: conventional RT plus concurrent chemotherapy and adjuvant chemotherapy. Concurrent chemotherapy consisted of mitomycin C at 10 mg/m^2 ^was administered on days 1 and 29, and oral 5-FU at 300 mg/day was administered on days 1–14 and 29–42 during RT. Adjuvant chemotherapy of 5-FU orally at 200 mg/day was given for three courses of 4 weeks, with a 2-week rest every 6 weeks. At a median follow-up time of 89 months, 5-year actuarial disease-free survival was 48.2, 54.1, 64.5, and 59.7% for arms 1, 2, 3, and 4, respectively. Pattern of failure revealed a significant increase in loco-regional recurrence but no distant metastases in the non-concurrent chemoradiotherapy arm: local recurrence was 25.5, 20.6, 14.3, and 17.6% for arms 1, 2, 3, and 4, respectively. Regarding tolerability, treatment was well-tolerated although acute side effects were generally higher in concurrent arms [54]. Although overall survival rates were not reported, the study supports the efficacy of this combination in treatment of locally advanced cervical cancer: nonetheless, the role of 5-fluorouracil is disputable due to the unpredictable bioavailability of this agent when administered by oral route [55].

The larger randomized study with mitomycin C as a single agent plus radiation in cervical cancer was published as an interim analysis immediately after the five randomized cisplatin-based studies. In this multicentric randomized phase III study, patients with locally advanced carcinoma were randomized to radiation alone (78 patients) or mitomycin C at 15 mg/m^2 ^(82 patients) on the first and sixth weeks of radiotherapy. Although 4-year survival was not statistically different, disease-free survival favored the mitomycin- containing arm (71 vs. 44%), as well as local and systemic control rates. On sub-group analysis, differences in disease-free survival were of greater magnitude in more advanced stages. Hematological and non-hematological toxicity were minimal, and there was no increase in acute radiation reactions. The trial continues to accrue patients and follow-up data [56]. Despite the fact that mitomycin C clearly possesses a role as radiosensitizer in cervical cancer treatment, there exist concerns regarding its significant toxicity. Christie et al. reported in their observations the toxicity of a series of 177 patients who also received chemotherapy that consisted of infusional 5-fluorouracil during the first and last weeks of the radiotherapy's external beam component, combined with bolus mitomycin C (64 patients) or without mitomycin C (29 patients). Median follow-up was 7.2 years, and median survival time was significantly higher for patients receiving mitomycin C; nevertheless, there was a relatively high complications rate, with 36% of patients having grade 3 or 4 complications that persisted through throughout follow-up time intervals. These data led the authors to conclude that use of mitomycin C in addition to radiotherapy and 5-fluorouracil should be regarded with caution [57].

In another publication, serious (grade 3) late bowel toxicity incidence in 154 patients with locally advanced cervical cancer entered in six consecutive chemoradiotherapy protocols between February 1982 and June 1987 was reported. Of these 154 patients, 54 patients were treated with mitomycin C, 5-fluorouracil, and radiation and were compared with 100 patients who received similar treatment without mitomycin C. Overall incidence of serious late bowel toxicity was 15.6%: 14 of 54 (26%) vs. 10 of 100 (10%) for patients who did or did not receive mitomycin C, respectively (*p *= 0.009). In multivariate analysis, administration of mitomycin C was the only factor predictive for late complication development (*p *= 0.012, odds ratio (OR) = 3.15; 95% confidence interval [95% CI], 1.3–7.7). Interestingly, significant reduction in late bowel toxicity was observed with elimination of mitomycin C from the chemoradiation protocols despite dose escalation of both radiation and 5-FU [58].

In anal cancer, after Nigro's report in 1974 demonstrating that anal carcinoma could be cured without the morbidity and functional consequences of an abdominoperineal resection [59], concurrent 5-fluorouracil and mitomycin-C was established as the standard for several randomized trials; however, substituting cisplatin for mitomycin C yields high rates of survival and sphincter preservation without the toxicity typically observed with mitomycin C use [60]; hence, use of cisplatin and 5-fluorouracil in treatment of patients with anal carcinoma is more widely accepted. In cervical cancer, the first evidence of the superiority of the combined treatment obtained from studies utilizing cisplatin-based therapies; thus, the role of mitomycin C either alone or in combination with 5-fluorouracil is limited.

### Epirubicin

4-Epidoxorubicin, an analog of anthracycline, is known to have activity against cervical cancer when tested as single agent in patients with recurrent or advanced disease [61,62]. While doxorubicin has been evaluated as a radiosensitizer in several systems including cervical cancer cell lines, demonstrating its synergy with radiation [63], there is a scarcity of studies on this with epirubicin. Ban et al. demonstrated that simultaneous exposure of hamster lung cells to epirubicin and radiation results in a synergistic killing of cells [64]. Wong et al. reported the results of a randomized study in which 220 patients with bulky stages I, II, and III were randomized to receive either standard pelvic radiotherapy or chemoradiation (epirubicin 60 mg/m^2^) followed by adjuvant chemotherapy with epirubicin 90 mg/m^2 ^administered at 4-week intervals for five additional cycles. After a median follow-up time of 77 months, patients who received epirubicin had a statistically significant superior disease-free and overall survival; nevertheless, local disease control was similar in both treatment arms, suggesting that the benefit of adding epirubicin was likely due to the adjuvant component [65].

## Newer radiosensitizers

### Camptothecins: topotecan and irinotecan

Camptothecin is a naturally occurring cytotoxic alkaloid that has a unique intracellular target, topoisomerase I, a nuclear enzyme that reduces supercoiled DNA torsional stress during replication, recombination, transcription, and DNA repair. The two most frequently studied agents of this class are topotecan and irinotecan, which are synthetic analogs designed to facilitate parenteral administration of the active lactone form of the compound by introducing functional groups to enhance solubility. Topotecan and irinotecan are now well-established components in the chemotherapeutic management of several neoplasms. Topotecan has modest activity in patients previously treated with ovarian and small cell lung cancer and is currently approved for use in the U.S. as second-line therapy in these diseases. Irinotecan is a prodrug that undergoes enzymatic conversion to the biologically active metabolite 7-ethyl-10-hydroxy-camptothecin. It is currently treatment-of-choice in combination with fluoropyrimidines as first-line therapy for patients with advanced colorectal cancer or as a single agent after failure of 5-fluorouracil-based chemotherapy [66].

Camptothecins are potent radiation sensitizers, because DNA topoisomerase I recently has been established as a biochemical mediator of radiosensitization in cultured mammalian cells by camptothecin derivatives. Interestingly, this sensitization appears to be schedule-dependent, cell cycle phase-specific, and not strictly drug cytotoxicity-dependent [67]. Despite the fact that there are data arguing against topo I expression level as a critical determinant of cell sensitivity to camptothecin in unselected human cancer cell lines [68], topo I activity has been found elevated in primary cervical cancers compared with normal cervix; in addition, combined treatment of sub-or post-confluent CaSki cells with camptothecin and ionizing radiation results in additive killing of cells [69].

Clinical studies with these agents as radiosensitizers are limited in cervical cancer. In a phase I trial of topotecan administered with standard external-beam radiotherapy in advanced squamous cell carcinoma of the cervix, patients were treated with a starting dose of 0.5 mg/m^2 ^and escalated by 0.25 mg/m^2 ^daily for 5 days on days 1–5 and 22–26 concomitantly with radiotherapy. The three patients administered the higher dose presented no dose-limiting toxicity (grade 3 anemia in one case and grade 2 leukopenia in two cases). The authors conclude that topotecan is safe at 1 mg/m^2^. At a median follow-up of 20 months (range, 11–40 months), five of nine treated patients were reported without evidence of disease [70]. In another small study, the toxicity of concomitant topotecan with scheduled brachytherapy after external radiation was evaluated within a phase I study. Patients received i.v. topotecan during their low-dose rate brachytherapy. The initial dose of topotecan was 0.5 mg/m^2^/day for 5 days concomitant with low-dose rate brachytherapy for two brachytherapy applications. No major toxicity was noted at this dose level in the initial three patients; however, at the 1 mg/m^2 ^dose, two patients experienced grade-4 and one, grade-3 hematological toxicity. Thus, the recommended dose of topotecan during low-dose rate brachytherapy was 0.5 mg/m^2 ^[71]. Currently, a phase I study (GOG-9913 and NCT00054444) of the combination of topotecan and cisplatin plus standard radiation is ongoing.

Regarding irinotecan, its activity in patients with cervical cancer has been demonstrated as a single agent and in combination with cisplatin in the recurrent and metastatic setting [72,73]; nonetheless, no clinical studies of its use as radiosensitizer have been reported to date. Remarkably, a preclinical study performed in the radiosensitive human cervical squamous cell carcinoma cell line ME180 with SN38, the active metabolite of irinotecan not only failed to show SN38 radiosensitizing properties, but also failed to radiosensitize SN38-resistant subclones established from ME180 cells [74]. Thus, current data suggest that irinotecan is not a promising agent for use in chemoradiation protocols for cervix carcinoma, but further preclinical studies on the issue are warranted.

### Vinorelbine

Vinorelbine, a semi-synthetic vinca alkaloid, is a potent inhibitor of mitotic microtubule polymerization. Its radiosensitizing properties were first evaluated in human lung carcinoma cell lines NCI-H460 and A549. In this system, pretreatment of NCI-H460 cells with vinorelbine for 24 h and then radiation potentiated the radiation effect in a dose-dependent manner, with the ratio of fractional survival with radiation to fractional survival (vinorelbine plus radiation) ranging from 1.7:1 at 1 Gy to 5.5:1 at 6 Gy. In this cell line, radiation induced a block in the G2/M phase of the cell cycle that peaked 10 h after treatment. Interestingly, the effect on this system was dependent on G2/M arrest induction. As in A549 cells in which radiation induced G1 block, vinorelbine was unable to potentiate the radiation effects [75]. Likewise, in another lung cancer cell line Fukuoka et al. demonstrated that PC-9 cells were sensitized to radiation by vinorelbine with a maximal sensitizer enhancement ratio at a 10% cell-survival level of 1.35 after 24-h exposure to vinorelbine at 20 nM. In addition, by flow cytometry the authors showed prolonged G2/M accumulation concomitant with continuous polyploidization and induction of vinorelbine-treated apoptosis cells that were then exposed to radiation [76]. It appears, however, that G2/M block is not necessary for inducing radiosensitizing. A study has demonstrated that with vinorelbine at 1 nM, the radiation-induced DNA strand breaks observed in human SCLC SBC-3 cells are not completely repaired at 24 hours suggesting that radiosensitization by vinorelbine may-at least in part-be associated with DNA repair impairment following radiation-induced DNA damage [77].

The sole study with vinorelbine as radiosensitizer was performed in patients with advanced cervical cancer and other pelvic malignancies in the context of a phase I trial. This study has two phases; in the first, vinorelbine was administered at a starting dose of 10 mg/m^2^/week with subsequent cohorts escalated in 5-mg/m^2^/week increments during external beam radiation. In 26 evaluable patients, vinorelbine at 25 mg/m^2^/week was well- tolerated the primary toxicity hematological. In the second part of the study, paclitaxel at a starting dose of 20 mg/m^2 ^was added to vinorelbine at 20 mg/m^2 ^and pelvic radiation; however, this combination was not tolerated. Five of six patients (83%) experienced leukopenia grade 2 or higher; thus, the investigators considered this combination not suitable for future trials [78].

### Paclitaxel

Paclitaxel is a potent microtubule-stabilizing agent that selectively blocks cells in cell-cycle G2 and M phases and is cytotoxic in a time concentration-dependent manner. Earlier investigations based on the radiobiological principle that G2 and M are the most radiosensitive cell-cycle phases suggested that paclitaxel could function as a cell cycle- selective radiosensitizer, which was demonstrated in an astrocytoma cell line [78]. The effect was evaluated in additional human cancer cell lines, demonstrating that paclitaxel can radiosensitize, to a modest degree, some but not all human cell lines by a mechanism that requires G2/M cell cycle block production [79]. The radiosensitizing effect of paclitaxel upon cervical cancer cell lines, however, has repeatedly been reported as inexistent or at best modest. Rave-Frank et al., on studying CasKi cells, reported that paclitaxel exerted a weak, non-statistically significant radiosensitizing effect on cervical carcinoma cells [80]. In another report studying ME180, SiHa, and MS751 cervical cancer cell lines, paclitaxel and radiation were supra-additive in two lines but subadditive in MS751, leading authors to conclude that the modest radiation-sensitizing effects in cervical cancer cell lines of this agent existed [81]. Similar findings were reported on lack of sensitation by paclitaxel utilizing C33A and MS751 cervical cancer cell lines under conventional fraction-size doses or radiation [82]. Another report on both taxanes-paclitaxel and docetaxel-actually found a reduction in radiosensitivity (up to a 3.3-fold reduction relative to radiation alone) in HeLa cells over a wide range of drug concentrations of these by means of a supra-additive, radiation-drug interaction was observed at drug concentrations above IC50 [83]. All together, these data suggest that paclitaxel would have a limited benefit as a radiosensitizer for cervical cancer treatment.

Despite this preclinical information, paclitaxel either alone or in combination with other agents has undergone evaluation as sensitizer in cervical cancer. There are no reports on formal phase I dose-finding studies of single agent paclitaxel as radiosensitizer in patients with cervical cancer. However, a pilot study of concurrent radiotherapy and weekly paclitaxel for locally advanced or recurrent squamous cell carcinoma of the uterine cervix was reported by an Italian group. The authors administered paclitaxel weekly at 40 or 60 mg/m^2 ^during the entire external radiotherapy course. Nineteen patients were evaluable for response; a complete response was obtained in eight of the 13 new cases (62%) and in four of the six recurrences (66%), for a total complete response rate of 63%. Regarding toxicity, five patients (26%) had grade 3 small bowel toxicity (three at 40 mg/m^2 ^and two at 60 mg/m^2^); another patient at 40 mg/m^2 ^presented grade 3 bladder toxicity, while another had grade 4 mucositis [84]. These data are difficult to interpret with regard to the recommended dose of paclitaxel in this setting. 

Three dose-finding studies have been performed combining either carboplatin or cisplatin with paclitaxel.  The first study was intended to determine the tolerable doses and potential toxicities of taxol, administered weekly, with concomitant cisplatin and radiation therapy in advanced cervical cancer. Paclitaxel was administered weekly as a 3-h i.v. infusion at a starting dose of 10 mg/m^2^/week and escalated to 10-mg/m^2^/week increments if tolerated by successive cohorts of three new patients, whereas cisplatin was administered every 3 weeks at 50 mg/m^2^. A total of 16 patients and 102 treatment cycles were evaluated. Dose escalation of taxol from 10–50 mg/m^2^/week was well-tolerated; there were no grade 3 episodes of toxicity with the exception of one grade 3 neutropenia. Other toxicities were mild. The authors suggest that 50 mg/m^2 ^of paclitaxel every week during external radiation concurrent with cisplatin at 50 mg/m^2 ^every 21 days is safe and effective in these patients [85].

Later, a second phase I study was reported in which 18 patients with cervical cancer received a fixed dose of cisplatin at 30 mg/m^2 ^and paclitaxel starting at 40 mg/m^2 ^with a 5-mg/m^2 ^escalation per level weekly for six times. Radiotherapy was administered to the pelvis with a four-field box technique for 5 days each week in 1.8 Gy fractions. Cohorts of three patients were enrolled at each level and three additional patients were included if one or two dose-limiting severe adverse events were recorded. Dose-limiting toxicity was defined as grade 3 or 4 non-hematological toxicity, excluding nausea or vomiting and alopecia, grade 4 neutropenia, or thrombocytopenia, and prolonged (>1 week) neutropenia or thrombocytopenia. Three, five, four, and six patients were enrolled at paclitaxel levels of 40, 45, 50, 55 mg/m^2^, respectively. Diarrhea was the dose-limiting side effect and maximal tolerated dose was paclitaxel at 50 mg/m^2 ^in this regimen of weekly cisplatin at 30 mg/m^2^. Overall response rate was 92.3%, suggesting that this scheme is effective for cervical cancer treatment [86].

Finally, a third study was conducted to determine the maximum tolerated dose (MTD) and dose-limiting toxicity (DLT) of weekly paclitaxel/carboplatin chemoradiotherapy in locally advanced cervix cancer with primary, previously untreated, squamous cell or adenocarcinoma of the cervix. In this study, paclitaxel was used at a fixed dose of 50 mg/m^2 ^and carboplatin was escalated to 0.5 AUC (starting at 1.5 AUC). A total of 15 patients were entered into the study and received a median number of seven courses; main toxicity was hematological and maximum tolerated dose of carboplatin was 2.5 AUC with 50 mg/m^2 ^of paclitaxel. Responses were encouraging, with estimated progression-free and overall survival of 80 and 86%, respectively [87].

### Gemcitabine

Gemcitabine, 2',2'-difluorodeoxycytidine, is a very potent and specific deoxycytidine analog that has been evaluated in combination with concurrent radiation for treatment of various types of cancer, demonstrating its safety within the range 100–1000 mg/m^2^/wk when used concurrently with radiation [88]. Gemcitabine radiosensitizes a wide variety of human cancer cell lines. Radiosensitization can be produced by either long exposure to a low concentration of gemcitabine or by a brief treatment with a higher but clinically relevant concentration. The effect occurs under conditions in which cells demonstrate concurrent redistribution into S phase and deoxyadenosine triphosphate pool depletion [89]. To date, it has been shown that gemcitabine is highly synergistic to radiation and cisplatin in cervical cancer cell lines [90,91] and produces responses >80% when used as neoadjuvant therapy combined with either cisplatin or oxaliplatin [92,93].

The highly radiosensitizing property of this antimetabolite led to its testing in cervical cancer. As a single agent, the first study performed reported in abstract form was performed by McCormack et al. in 10 previously untreated patients with stage IB2-IIIB cervical cancer. All patients underwent external beam radiotherapy; 50.4 Gy was given in 28 fractions over 5.5 weeks plus high-dose-rate intracavitary brachytherapy. The starting dose of gemcitabine was 50 mg/m^2 ^up to 150 mg/m^2^. At these doses, gemcitabine was well-tolerated; no patient experienced severe toxicity but all presented mild-to-moderate diarrhea, and only three presented mild myelosuppression; thus, no dose-limiting toxicities were found. It is of note that all but one patient were reported alive and disease-free at a median follow-up of 29 months (range, 9–33 months), which strongly supported preclinical data regarding its potent radiosensitizer properties [94]. Based on these data, Pattaranutaporn et al. reported the results of a phase II trial in which they chose the dose of 300 mg/m^2 ^weekly for 5 weeks during external radiation, which was delivered in 2-Gy fractions for 50 Gy for treatment of FIGO stage IIIB patients. At this dose, gemcitabine was well-tolerated, with reports of only a single patient with grade 3 diarrhea and anemia; grade 4 toxicities were not reported. It is noteworthy that all but two patients achieved a complete response and at a median follow-up of 20 months, disease-free and overall survival were 84 and 100%, respectively [95]. An additional small study of gemcitabine as single agent was reported by Boulaga et al., who treated 19 patients with locally advanced cervical cancer with a scheme consisting of gemcitabine 300–600 mg/m^2 ^administered on days 1, 8, 15, 40, and 47 together with external beam pelvic radiotherapy (45 Gy over 4.5 weeks, followed by a 20-Gy boost after 1 week of rest); again, no grade 3 or 4 toxicities were encountered, and 80% of patients achieved complete response [96].

The high activity and tolerability of gemcitabine during radiation was also reported in patients suffering from ureteral tumor obstruction-associated renal dysfunction. In this report, eight cervical carcinoma patients whose serum creatinine ranged from 1.6–18.5 mg/100 mL (median, 3.3; mean, 6.8) received gemcitabine at 300 mg/m^2 ^concurrent with standard pelvic radiation. Despite the fact that the majority of patients had grade 3 leukopenia and neutropenia, dermatitis, colitis, and proctitis, eight of nine (89%) patients achieved complete response and all exhibited improvement in creatinine clearance (pre-therapy, 22.78; post-therapy, 54.3 mg/ml/min) (*p *= 0.0058) and all but one normalized serum creatinine. At a median follow-up of 11 months (range, 6–14 months), all patients are alive, one with pelvic and another with systemic disease. The authors suggest that ureteral obstruction causing any degree of renal insufficiency should not be a contraindication for receiving chemoradiation to attempt a cure and that in this setting in which cisplatin is contraindicated; gemcitabine use should be considered [97].

Because gemcitabine synergizes not only radiation but also cisplatin, investigators proceeded to evaluate the combination of cisplatin and gemcitabine concurrent with radiation. Alvarez et al. performed a feasibility study utilizing a biweekly regimen of cisplatin at 30 mg/m^2 ^and gemcitabine at 20 mg/m^2^. External beam radiation was delivered to the entire pelvic region in 23 fractions over 5 weeks for a total dose of 46 Gy. In addition, two brachytherapy insertions (total dose, 85–90 Gy at point A) were administered at the third and fifth weeks. This planned scheme proved to be toxic, because the first three patients presented grade 3 or higher hematological toxicity; hence, cisplatin was administered only once a week. At this level, grade 3 or 4 hematological toxicities were maintained <10%. Regarding the efficacy of this combination, complete response rate in the 37 evaluable patients was 86% [98,99]. These studies with the combination of cisplatin and gemcitabine plus radiation were not intended, however, to establish the recommended dose for the scheme.

Zarba et al. reported a phase I-II study with the aim to establish the recommended weekly dose of gemcitabine to be used with the "standard" dose of cisplatin (40 mg/m^2^) during radiation therapy. In their study, patients with locally advanced cervical cancer were to be treated with cisplatin at 40 mg/m^2 ^plus escalating doses of gemcitabine beginning at 75 mg/m^2 ^with 25-mg/m^2 ^increments. Radiation consisted of 50.4 Gy in fractionated doses over 5 weeks, followed by brachytherapy at 30–35 Gy delivered to point A. Results of the first part of the study show that the recommended weekly dose of gemcitabine to be used with cisplatin was 125 mg/m^2^. At this level, grade 3 toxicity was principally non-hematological and included diarrhea (21%), mucositis (13%), nausea/vomiting (13%), skin toxicity (13%), and asthenia (4%); the only grade 4 toxicity was neutropenia, occurring in a single patient (4%). Similar to other studies conducted with the combination, complete response rate in the 36 evaluable patients was 89%, and at a median follow-up of 14 months 81% of the total study population were reported as disease-free [100].

Despite these encouraging results with gemcitabine and particularly in combination with cisplatin, there was no evidence on the superiority of any combination of radiosensitizers over cisplatin alone. In this line, a phase II randomized study was initiated primarily to compare rate of pathologic complete response as a surrogate marker of survival [101] between the experimental arm of cisplatin and gemcitabine using the Zarba regimen (40 mg/m^2 ^and 125 mg/m^2^, respectively, vs. cisplatin alone (40 mg/m^2^). In this study, patients staged as IB2, IIA, and IIB received six weekly courses of one of the two schemes during external beam radiation and, within 3 weeks after radiation, patients were taken to radical hysterectomy with pelvic and para-aortic lymphadenectomy. Brachytherapy in this study was only administered in the adjuvant setting to cases with any intermediate or high-risk factor for recurrence (intermediate risk, vascular or lymphatic permeation, deep stromal invasion, or residual tumor <2 cm; high-risk: pelvic lymph node positivity, close or positive surgical margins, or disease in parametria). In addition, patients in whom pathologic analysis of resected nodes found positive para-aortic nodes were scheduled to receive cisplatin and radiation to the para-aortic field. The results of this study are quite promising: 83 patients were evaluable for toxicity and 80 for response. Complete pathologic response in the cisplatin group was 55%, (95% CI, 35.5–73) and 77.5% (95% CI, 57–90) for the gemcitabine cisplatin arm (*p *= 0.0201). Among partial responders, there were seven patients in the cisplatin arm with high, and seven with high-intermediate, risk factors for recurrence in their surgical specimens vs. two and three cases with these characteristics, respectively, in the gemcitabine-cisplatin arm. These results were observed despite the fact that the combination regimen was more toxic (gastrointestinal and hematological toxicity) and had fewer weekly doses delivered and lower cisplatin dose-intensity, which resulted in a longer time to complete external radiation in the experimental arm [102].

These results strongly supported the high efficacy of this combination and led to the design of a multicenter, open-label, randomized phase III study in which 500 evaluable, FIGO stage IIB-IVA patients were randomized to the experimental arm, consisting of cisplatin at 40 mg/m^2 ^and gemcitabine at 125 mg/m^2 ^during external beam radiation followed by brachytherapy plus two courses of adjuvant cisplatin at 75 mg/m^2 ^d1 and gemcitabine 1000 mg/m^2 ^d1 & d8 or to the control arm of cisplatin chemoradiation at 40 mg/m^2 ^with no adjuvant therapy. The study is closed and results are pending.

On the other hand, the remarkable high pathologic response rate obtained with the combination of gemcitabine and cisplatin chemoradiation without brachytherapy led the authors to test whether brachytherapy could be dispensable in the setting of a highly active chemoradiation; hence, a randomized phase III study was initiated in which FIGO stages IB2-IIB patients are allocated to brachytherapy or radical hysterectomy with pelvic and para-aortic lymphadenectomy after pelvic external beam radiation plus cisplatin and gemcitabine is delivered in both arms of treatment [ISRCTN88773338].

Recently, the Puget Sound Oncology Consortium reported the results of a phase I study aimed at defining the maximum tolerated dose of weekly gemcitabine administered concomitantly with standard weekly cisplatin and pelvic radiotherapy for primary treatment of cervical cancer. Different from the previously mentioned studies of cisplatin and gemcitabine in combination in which cisplatin was administered first, in this study the inverse order was chosen; thus, gemcitabine was followed by cisplatin. Cisplatin was administered at a fixed dose of 40 mg/m^2 ^and gemcitabine dose initiated at 100 mg/m^2^. Radiation consisted of 45–50 Gy in 25 daily fractions combined with brachytherapy to deliver at least 85 Gy at point A. Surprisingly, three of the six patients receiving gemcitabine at 100 mg/m^2 ^presented dose-limiting toxicity that consisted of severe fatigue, lymphopenia, diarrhea, and tinnitus; therefore, de-escalation to 50 mg/m^2 ^was decided upon. At this dose, both of the two treated patients also had dose-limiting toxicity; therefore, the study was halted. Despite the fact that all patients achieved complete response, the tested schedule produced unacceptable toxicity, which led the authors to conclude that gemcitabine-administered prior to cisplatin with radiation for cervical cancer-will likely require cisplatin dose reduction [103]. Whether this schedule of gemcitabine first is or is not more effective will require additional testing. The results of another study of cisplatin-gemcitabine with the Zarba regimen (cisplatin first) have just been published. In this study, external beam pelvic radiation was delivered 5 days a week for a total of 50 Gy in 25 fractions over 5 weeks. After completion of external radiation, patients received brachytherapy with Cesium-137 via standard Fletcher-suit applicators, delivering 30 Gy to point A. Twenty of 23 enrolled patients completed the treatment and were evaluable for response and toxicity. Complete response rate was 90% (18/20), and toxicity was moderate: two patients required blood transfusions; 5% of patients had grade 2 leukopenia or thrombocytopenia; 40% had grade 1–2 nausea/vomiting, and 50% had grade 1 diarrhea. At a median follow-up of 12 months, all patients are alive and 16/20 (80%) are disease-free [104]. Taken together, these results clearly position gemcitabine as one of the most promising radiosensitizer agents for cervical cancer. Results of the multicenter randomized trial comparing cisplatin vs. cisplatin-gemcitabine are eagerly awaited.

### Capecitabine

Capecitabine is an orally available fuoropyrimidine carbamate that generates the active drug 5-Fura selectively in tumors by three enzymes located in liver and in tumors; the final step is the conversion of the intermediate metabolite 5'-dFUrd into 5-FUra by dThdPase (thymidine phosphorylase) in tumors [105]. This conversion appears to be a rate-limiting step for capecitabine efficacy, as it has been observed that conversion was insufficient in a human cancer xenograft line, which was refractory to capecitabine in *in vivo *therapy, and that human cancer xenograft susceptibility to 5'-dFUrd correlated with their dThdPase expression levels [106]. Thymidine phosphorylase is expressed in cervical carcinoma cell lines and in a xenograft model; this enzyme has been induced by radiation, enhancing the cytotoxicity of radiation and capecitabine [107,108]. In addition to mechanistic bases, the advantages in using an oral agent for chemoradiation appear obvious; hence, capecitabine has been used either alone or in combination with cisplatin for radiosensitization in cervical cancer. Torrecillas et al. reported the preliminary results of a phase I study of capecitabine concurrent with standard pelvic radiation for the primary treatment of locally advanced cervical cancer. They found 825 mg/m^2 ^twice daily for 5 days a week for 6 weeks as the dose previously used for phase II trials [109]. This dose level is within the range reported for capecitabine and pelvic radiotherapy for patients with rectal cancer, which varies from 1,600–1,800 mg/m^2 ^[110,111]. Capecitabine has proven superior to 5-fluorouracil in terms of response rate for metastatic colorectal cancer [112]; therefore, its use in combination with cisplatin and radiation for cervical cancer would eventually yield better results than those obtained with cisplatin and 5-fluorouracil. To date, the results of a dose-escalation study of this drug combination during radiation has been reported in patients with locally advanced cervical cancer. In this study, 13 patients received a twice-daily dose of capecitabine at either 300 or 450 mg/m^2 ^plus a fixed dose of weekly cisplatin at 40 mg/m^2 ^during external pelvic radiation. Main toxicities were hematological and gastrointestinal. Remarkably, three patients (23%) presented late Radiation Therapy Oncology Group/Eastern Cooperative Oncology Group (RTOG/ECOG) grade 3 toxicity bladder or vaginal mucosa at 6, 9, and 15 months. Thus, the maximum tolerated dose reported was 450 mg/m^2^; therefore, the authors recommended 300 mg/m^2 ^as the dose to be employed with cisplatin for future trials [113].

## Non-cytotoxic radiosensitizers

### Interferons and retinoids

It has long been known that interferons and retinoids have the ability to potentiate the effect of radiation in experimental systems either alone or combined [114,115]. Earlier clinical studies using the combination showed encouraging results. Lippman et al. reported on a phase II study in which 26 patients with untreated, locally advanced squamous cell carcinoma of the cervix were treated daily for at least 2 months with oral 13-cRA (1 mg/kg) and subcutaneous (s.c.) recombinant human IFN alpha-2a (6 million units), observing a response rate of 58% [116]. Subsequent studies in pre-treated cervical cancer failed to demonstrate the antitumor activity of the combination [117,118]. Nevertheless, a pilot study was conducted to evaluate the clinical efficacy and tolerability of the interferon-alpha 2a, 13-cis-retinoic acid combination and radiotherapy; although the treatment was well-tolerated, the clinical response rate was only 47% [119]. In line with these results, a small, underpowered randomized trial of interferon alpha-2b added to standard radiation therapy vs. only radiation did not significantly improve loco-regional response or survival [120]. These results argue against the value of this combination of biological modifiers used as radiosensitizers for cervical cancer treatment.

### Hyperthermia

Hyperthermia has shown to enhance the effects of radiation in a variety of experimental systems [121,122]. Based upon the biologic rationale and in view of the recent advances in heating and thermometry techniques, radiotherapy in combination with hyperthermia has been tested in cervical cancer. In a small randomized study performed in FIGO stage IIIB cervical cancer, 40 patients were treated with either radiation alone, which consisted of external beam irradiation to the pelvis combined with Iridium 192 high-dose-rate intracavitary brachytherapy, or with the same radiation plus three hyperthermia sessions. The primary end-point of this study comprised local complete response and survival. Results showed a complete response rate of 50% (10 of 20) in the radiation group vs. 80% (16 of 20) in the thermoradiation group, this difference statistically significant (*p *= 0.048); however, differences in 3-year overall survival and disease-free survival were not statistically significant between the treatment arms [123]. This study was unpowered, and therefore conclusions must be taken with caution; however, the International Atomic Energy Agency sponsored a multi-institutional, prospective randomized trial in 110 patients with locally advanced cervical cancer to evaluate the effect on local control of hyperthermia during radiation. Patients received external beam radiation therapy and brachytherapy with or without hyperthermia, with a minimum of five sessions (60 min each, once a week) employing a radiofrequency-capacitive heating device. Intratumoral temperature was measured at the first hyperthermic treatment, and at least once more during the treatment course. Results demonstrated that patients in the two arms were well-balanced with regard to clinical characteristics. At a median follow-up time of 15 months, there were no differences in either local control or survival; however, acute grade 2–3 toxicity frequency was significantly higher as observed in patients receiving hyperthermia (18 vs. 4%). Sub-group analyses showed worse survival in hyperthermia-group patients staged as IIB despite a similar local control rate [124]. These results suggest a possible negative influence of this form of therapy that requires further investigation.

### Molecular-targeted therapies as radiosensitizers

Irradiation evokes a plethora of cell responses through several pathways, including those involved in cell proliferation, cell cycle regulation, apoptosis, angiogenesis, and inflammation. The current preclinical and clinical availability of a number of newer products collectively termed "molecular targeted agents" has led to their study as new forms of radiosensitization; nonetheless, this interesting topic is beyond the scope of the present work. Comprehensive reviews on that are already being published [125-129]; hence, we limit ourselves herein to list molecular-targeted therapeuticals that have demonstrated potential to be used as radiosensitizers for the treatment of solid tumors (Table 4) and to ongoing clinical trials evaluating these drugs as radiosensitizers in cervical cancer (Table 5).

## Conclusion

Experimental evidence shows that the majority of current cytotoxic agents widely used in clinical oncology practice are indeed radiosensitizers. Recently, chemoradiation trials proved the efficacy and good toxicity profile of cisplatin, and positioned it as the standard agent to be used to sensitize cancer cells to radiation in cervical cancer treatment. Thus, older agents such as mitomycin C and even 5-fluorouracil have played a limited role in this malignancy. Newer cytotoxic agents with radiosensitizing properties, such as topotecan, vinorelbine, paclitaxel, capecitabine, and gemcitabine, have demonstrated promising activity either alone or in combination with cisplatin in phase I studies or small phase II studies. In this line, gemcitabine is the newer cytotoxic agent with the most extensive evaluation. A randomized phase II trial demonstrated the superiority of the combination of standard cisplatin plus gemcitabine over cisplatin alone in terms of pathologic complete response rate, and an ongoing phase III trial that has completed accrual will eventually confirm these results regarding survival. On the other hand, we wish to underscore that several molecular targeted agents possessing radiosensitizing properties open the way for their testing either alone or with known cytotoxic radiosensitizers for cervical cancer. Among the latter, cetuximab, a monoclonal antibody against the epidermal growth factor receptor, and a combination of epigenetic therapy agents are being tested in patients with cervical cancer as an adjunct to chemoradiation with cisplatin.

## Competing interests

The author(s) declare they have no competing interests.

## Authors' contributions

MC, AG and LC compiled information and critically reviewed the manuscript. AD-G conceived of and wrote the manuscript.

**Table 1:** Phase 1 studies of newer radiosensitizers including platinum compounds

**Agent [ref]**	**Radiation**	**Dose**
CDDP (daily) [29]	EBRT	8 mg/m^2^
Carboplatin (CI) [37]	EBRT	12 mg/m^2^
Carboplatin (weekly) [39]	EBRT	133 mg/m^2^
Topotecan daily on days 1–5 & 22–26 [70]	EBRT	1 mg/m^2^
Topotecan daily on days 1–5 [71]	Brachy	0.5 mg/m^2^
Topotecan plus CDDP	EBRT	no results yet
Irinotecan plus CDDP	EBRT	no results yet
Vinorelbine (weekly) [78]* Vinorelbine + paclitaxel	EBRT	25 mg/m^2 ^to toxic
Paclitaxel (weekly) + CDDP (every 21 days) [85]	EBRT	50 mg/m^2 ^and 50 mg/m^2^
Paclitaxel (weekly) + CDDP (weekly) [86]	EBRT	50 mg/m^2 ^and 30 mg/m^2^
Paclitaxel (weekly) +Carboplatin (weekly) [87]	EBRT	50 mg/m^2 ^and 2.5 AUC
Gemcitabine (weekly) [94]	EBRT	DLT not reached at 150 mg/m^2^
CDDP (weekly) +Gemcitabine (weekly) [100]	EBRT	40 mg/m^2 ^and 125 mg/m^2^
Gemcitabine first (weekly) +CDDP (weekly) [103]	EBRT	50 mg/m^2 ^and 40 mg/m^2 ^to toxic
Capecitabine (twice daily) [109]	EBRT	825 mg/m^2^
Capecitabine +CDDP (twice daily) [113]	EBRT	825 mg/m^2^

*To toxic when used with paclitaxel at 20mg/m2.

**Table 2:** Phase 2 studies of newer radiosensitizers alone or combined

**Schedule [ref]**	**# pat**	**Complete R**	**Survival**
Gemcitabine 300 mg/m^2 ^(weekly) [95]	--	89%	DFS 84%* OS 100%
Gemcitabine 300–600 mg/m^2 ^days 1, 8, 15, 40, and 47 [96]	19	80%	NR
Gemcitabine 300 mg/m^2 ^(weekly) [97]	9	89%	DFS 77%** OS 100%
CDDP 30 mg/m^2 ^+ Gemcitabine 20 mg/m^2 ^(both biweekly) [98,99]	37	86%	NR***
CDDP 40 mg/m^2 ^+ Gemcitabine 125 mg/m^2 ^(both weekly) [100]	36	89%	DFS 81%°
CDDP 40 mg/m^2^	40	Path 55%	°°
Vs			
CDDP 40 mg/m^2^+ Gemcitabine 125 mg/m^2 ^(both arms weekly) [102]	43	Path 77.5%	
CDDP 40 mg/m^2 ^+ Gemcitabine 125 mg/m^2 ^both weekly) [104]	20	90%	DFS 80% OS 100%°°°

*Median follow-up 20 months. ** Median follow-up 11 months, trial in patients with renal failure. *** After 3 patients, CDDP was administered weekly. °Median follow-up 14 months. °° Randomized study. Patients underwent radical hysterectomy after EBRT. °°°Median follow-up 12 months.

**Table 3:** Ongoing phase 3 studies of newer radiosensitizers

**Arms**	**# Pat**	**Radiation**
CDDP 40 mg/m^2^	250	EBRT 50.4 + brachyterapy
Vs		
CDDP 40 mg/m^2^+ Gemcitabine 125 mg/m^2 ^+ Two post-brachy 21-day adjuvant courses CDDP 75 mg/m^2^/d1 plus Gemcitabine 1 gr/m^2 ^d1&d8	250	EBRT 50.4 + brachyterapy
CDDP 40 mg/m^2^+ Gemcitabine 125 mg/m^2^	180	EBRT 50 Gy in 2 Gy fractions+ Brachytherapy
Vs		
CDDP 40 mg/m^2^+ Gemcitabine 125 mg/m^2^	180	EBRT 50 Gy in 2 Gy fractions+ Radical hysterectomy

**Table 4:** Molecular targeted therapies with demonstrated preclinical activity as radiosensitizers

**Epidermal Growth Factor Receptor inhibitors**
Cetuximab
Gefitinib
Trastuzumab
CI-1033 (pan-ErbB tyrosine kinase inhibitor)
**Farnesyl Transferase Inhibitors**
FTI-277
L744832
BIM-46068
**Ras-Mediated Downstream Pathways inhibitors**
AS-ON Raf
PI3K inhibitor: LY294002
PI3K inhibitor: wortamannin
AS-ON p21
MEK inhibitor
**Agents that target apoptosis, cell cycle, NF-Kappa-B**
Flavopiridol
PS-341
UCN-01
**COX-2 Inhibitors**
SC-236
NC-398
Rofecoxib
**Antiangiogenic Agents**
mAb-VEGF
SU5416
SU5416
SU6668
mAb-VEGF-2
Angiostatin
Endostatin
**Histone deacetylase inhibitors**
Valproic acid
MS275
SAHA

**Table 5:** Ongoing protocols of molecular targeted therapies as radiosensitizers

**Phase 1**
-Cetuximab, cisplatin, and radiation therapy in treating patients with stage IB, stage II, stage III, or stage IVA cervical cancer (GOG-9918, NCT00104910)
-A phase I-II study of the COX-2 inhibitor celecoxib and chemoradiation in patients with locally advanced cervical cancer (RTOG C-0128)
**Phase 2**
-Radiation therapy plus celecoxib, fluorouracil, and cisplatin in patients with locally advanced cervical cancer. NCT00023660
-Phase II trial of the combination of DNA methylation inhibitor hydralazine and the histone deacetylase inhibitor magnesium valproate added to cisplatin chemoradiation in FIGO stage IIIB patients [128, 129].
